# AT_1_ receptor blockade alters nutritional and biometric development in obesity-resistant and obesity-prone rats submitted to a high fat diet

**DOI:** 10.3389/fpsyg.2014.00832

**Published:** 2014-07-29

**Authors:** Pauline M. Smith, Charles C. T. Hindmarch, David Murphy, Alastair V. Ferguson

**Affiliations:** ^1^Department of Biomedical and Molecular Sciences, Queen’s UniversityKingston, ON, Canada; ^2^The Henry Wellcome Laboratories for Integrative Neuroscience and Endocrinology, University of BristolBristol, UK; ^3^Department of Physiology, Faculty of Medicine, University of MalayaKuala Lumpur, Malaysia

**Keywords:** obesity, angiotensin, losartan, diet induced obesity, angiotensin receptor

## Abstract

Obesity is a chronic metabolic condition with important public health implications associated with numerous co-morbidities including cardiovascular disease, insulin resistance, and hypertension. The renin angiotensin system (RAS), best known for its involvement in cardiovascular control and body fluid homeostasis has, more recently, been implicated in regulation of energy balance. Interference with the RAS (genetically or pharmacologically) has been shown to influence body weight gain. In this study we investigated the effects of systemic AT_1_ receptor blockade using losartan on ingestive behaviors and weight gain in diet induced obese (DIO) rats. Prior to losartan administration (30 mg/kg/day) body weight gain remained constant within the DIO animals (3.6 ± 0.3 g/day, *n* = 8), diet resistant (DR) animals (2.1 ± 0.6 g/day, *n* = 8) and in the age-matched chow fed control (CHOW) animals (2.8 ± 0.3 g/day, *n* = 8), Losartan administration abolished body weight gain in animals fed a high fat diet (DIO: -0.4 ± 0.7 g/day, *n* = 8; and DR: -0.8 ± 0.3 g/day, *n* = 8) while chow fed animals continued to gain weight (2.2 ± 0.3 g/day, *n* = 8) as they had previously to oral administration of losartan. This decrease in daily body weight gain was accompanied by a decrease in food intake in the HFD fed animals. Following the removal of losartan, both the DIO and DR animals again showed daily increases in body weight gain and food intake which were similar to control values. Our data demonstrate that oral losartan administration attenuates body weight gain in animals fed a HFD whether the animal is obese (DIO) or not DR while having no effect on body weight gain in age-matched chow fed animals suggesting a protective effect of losartan against body weight gain while on a HFD.

## INTRODUCTION

Obesity is a chronic metabolic condition with important public health implications associated with numerous co-morbidities including cardiovascular disease, insulin resistance, and hypertension. Adipose tissue, once thought solely as a storage depot for excess triglycerides, is now known to be an important endocrine organ. Adipocytes produce and release a number of adipokines, which have been shown to act centrally to influence food intake and energy metabolism (see Woods et al., 1998; Galic et al., 2010; Harwood, 2012; Williams and Elmquist, 2012 for review).

The diet induced obesity (DIO) animal model is commonly used to study obesity and its numerous co-morbidities. In contrast to genetic models of obesity, DIO develops as a consequence of consuming a high fat diet (HFD) or high fat/high sugar diet for a period of time and, thus, more closely mimics the etiology of obesity in the majority of humans that display this phenotype. Interestingly, not all animals or people who prefer/consume the HFD become obese, and these subjects are described as diet resistant (DR; Levin et al., 1989; Smith and Ferguson, 2012). The reason for this divergence is unknown (Levin et al., 1989). The DIO phenotype is only partially explained by increased food intake (Levin et al., 1989); obese animals on a HFD diet develop a resistance to the actions of leptin (El-Haschimi et al., 2000; Lin et al., 2000; Wang et al., 2001; Boyle et al., 2011), an adipokine shown to be a key player in the control of food intake and energy metabolism (see Friedman and Halaas, 1998; Jequier, 2002; Galic et al., 2010 for review).

While the renin angiotensin system (RAS) is best known for its involvement in cardiovascular control and body fluid homeostasis, the RAS has more recently been implicated in the regulation of energy balance. Not only does adipose tissue have a local RAS (for review see Cassis et al., 2008; de Kloet et al., 2010; Frigolet et al., 2013) but serum levels of all the components of the RAS [renin, angiotensinogen (AGT), angiotensin (ANG) converting enzyme] are elevated in obesity (Cooper et al., 1998; Yasue et al., 2010). In addition, ANG receptors (Burson et al., 1994; Crandall et al., 1994; Cassis et al., 1996) and all the components of the RAS have been localized and been shown to be fully functional in adipose tissue (Cassis et al., 1988; Engeli et al., 1999; Fowler et al., 2009), and expression of these components has been shown to positively correlate with adiposity (Hainault et al., 2002; Engeli et al., 2005; see Kalupahana and Moustaid-Moussa, 2012 for review). The RAS has also been implicated in energy expenditure, a key component in energy balance. Recent studies have demonstrated that AGT deficient (*Agt*^-^/^-^) mice or mice lacking the AT1a receptor (*Agtr1a*^-^*/*^-^) have attenuated body weight and adiposity when fed a HFD (Massiera et al., 2001; Kouyama et al., 2005). These changes were accompanied by increased locomotor activity (Massiera et al., 2001) and rectal temperature and O_2_ expenditure (Kouyama et al., 2005). The fact that both the *Agt*^-^/^-^ and *Agtr1a*^-^*/*^-^ animals and their wild type counterparts consumed the same amount of the HFD suggests that attenuation of diet-induced weight gain and adiposity of the *Agt*^-^/^-^ and *Agtr1a*^-^*/*^-^ animals may be due to the increased energy expenditure. Lastly, pharmacological manipulations of the RAS have demonstrated an involvement for the RAS in body weight regulation and composition in both obese (Stucchi et al., 2009; Miesel et al., 2012; Premaratna et al., 2012) and non-obese animal models (Santos et al., 2008; Weisinger et al., 2008). As such, recent attention has been directed toward the use of antagonists of the RAS in the treatment of obesity (see Weisinger et al., 2009a for review).

In light of these observations, the present study was undertaken to determine the influence of AT_1_ receptor blockade on nutritional behavior and biometric development in obesity-resistant and obesity-prone rats submitted to a HFD.

## MATERIALS AND METHODS

All procedures were conducted in accordance with the Canadian Council on Animal Care regulations and approved by Queen’s University Animal Care Committee.

### DIET INDUCED OBESITY

Upon arrival, male Sprague–Dawley rats (125–150 g) were housed in pairs in a temperature controlled room on a 12 h light–dark cycle and exposed to either a HFD (Research Diets, New Brunswick, NJ #D12451, composition 45% kcal% fat, 35% kcal% carbohydrate, and 20% kcal% protein) or standard chow diet (LabDiet 5001, composition 13.5% kcal fat, 58% kcal carbohydrate, and 28.5% kcal% protein) with water provided *ad libitum*. Weight gain was measured on a weekly basis from time of arrival until week 10 at which time animals exposed to the HFD were divided into DIO or DR based on those who gained the greatest and least weight, respectively. Animals that were greater than 700 g were placed in the DIO group while animals that weighed less than 600 g were considered DR. Animals that were of intermediate weights (600–700 g) were eliminated from this study as they could not be reliably classified as DR or DIO rats. The DIO and DR phenotypes were validated in accordance with the DIO model of others (Levin and Dunn-Meynell, 2002; Hyland et al., 2007; Li et al., 2011) and in our own colony (Smith and Ferguson, 2012).

Rats were continued on their respective diets and body weight, food intake and water intake were measured daily between 8 am and 10 am from week 17. After a control period of 14 days, the ANG type 1 receptor (AT1R) antagonist, losartan (Sigma Chemical Company), was administered in the drinking water for 2 weeks at 30 mg/kg (Konno et al., 2012; Oliveira-Junior et al., 2014). Losartan concentration was calculated daily for each animal based on that day’s weight and the previous 24 h water consumption. Following the 2 week losartan treatment, animals were returned to normal tap water for an additional 10 days.

### DATA ANALYSIS

A one way analysis of variance (ANOVA) was used to determine if body weights were different between animals in the DIO, DR, and CHOW groups. Mean body weight gain, food intake/100 g body weight, and water intake/100 g body weight were obtained for each group (DIO, DR, and CHOW) for 7 days immediately prior to losartan administration, days 1–7 and days 8–14 of losartan treatment, and days 3–10 post losartan treatment. A repeated measures ANOVA and *post hoc* Tukey multiple comparison tests (*p* < 0.05 was considered as significant) was used to determine whether body weight gain, food intake, or water intake was altered as a result of losartan treatment in each of the three groups (DIO, DR, CHOW).

## RESULTS

**FIGURE 1:**
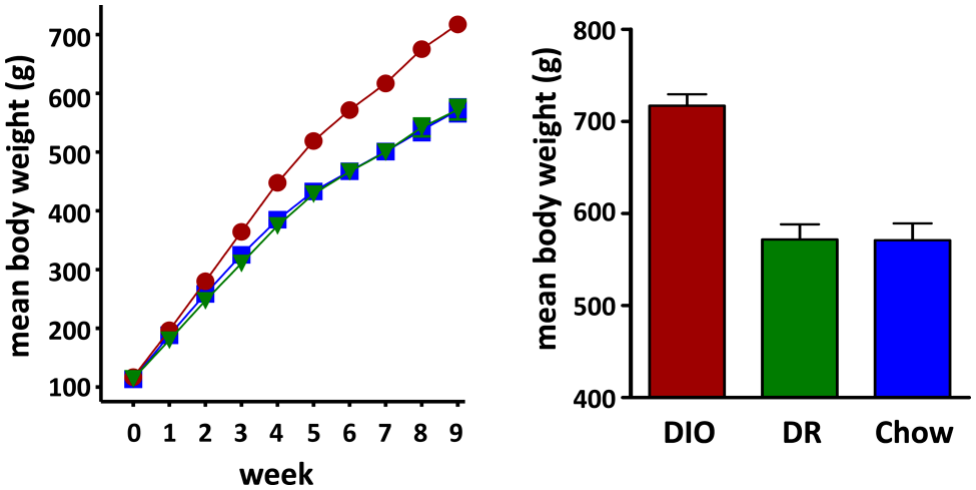
**Diet Induced Obesity (DIO) Phenotype: mean weekly body weight of rats classified as DIO (red circle) or diet resistant (DR, green triangle) after 8 weeks of *ad libitum* feeding on a HFD and age-matched chow fed controls (Chow, blue square) Bar graph on the right shows the mean body weights of rats considered DIO or DR based on those who gained the greatest and least weight, respectively, and age-matched chow fed control animals at time of group assignment**.

A total of 24 rats were used in this study of which 8 were in the DIO group, 8 were in the DR group and 8 animals were age-matched chow fed controls (CHOW). Rats classified as DIO, based on weight gain after 10 weeks on the HFD, had a mean body weight of 717.1 ± 12.4 g (*n* = 8). DR rats weighed the same as age-matched chow fed controls (DR mean body weight = 571.8 ± 16.5 g, *n* = 8; chow fed mean body weight = 570.9 ± 18.4 g, *n* = 8; ANOVA *p* < 0.0001, chow vs DR, ns Tukey *post hoc* analysis) and weighed significantly less than the rats classified as DIO (DIO vs DR, *p* < 0.001 Tukey *post hoc* analysis; see **Figure 1**).

At week 17, the beginning of daily body weight, food intake, and water intake measurements, DIO rats weighed significantly more (DIO 892.6 ± 11.3 g, *n* = 8, *p* < 0.001 one way ANOVA) than both the HFD fed DR rats (658.8 ± 16.4 g, *n* = 8, *p* < 0.05 Tukey *post hoc* analysis vs DIO) and chow fed control (CHOW) animals (692.5 ± 25.2 g, *n* = 8, *p* < 0.001 Tukey *post hoc* analysis vs DIO) while body weights of the DR and CHOW animals were not significantly different (*p* > 0.05 DR vs CHOW, Tukey *post hoc* analysis).

**FIGURE 2:**
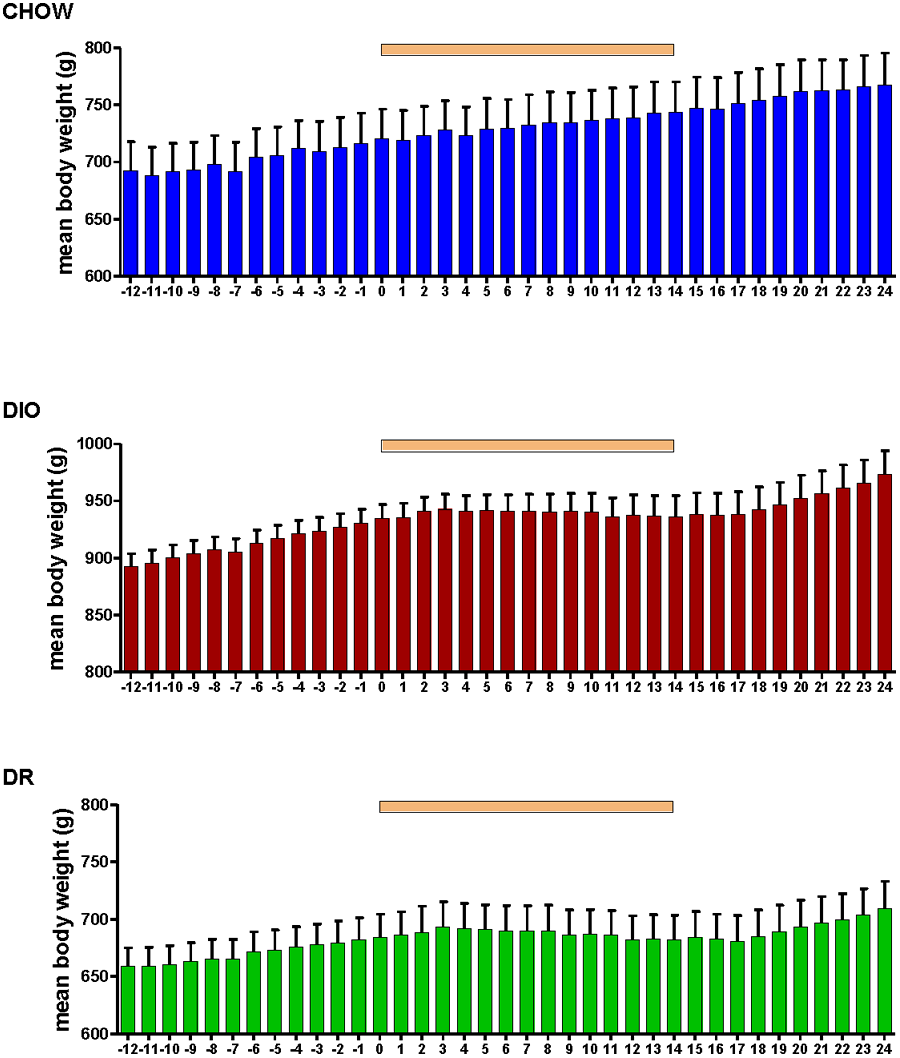
**Mean daily body weight in age-matched control rats fed standard rat chow (CHOW, upper panel), DIO rats (middle panel), and DR rats (lower panel) before, during and after oral administration of losartan.** Time of losartan administration indicated by the orange bar over the graphs.

**FIGURE 3:**
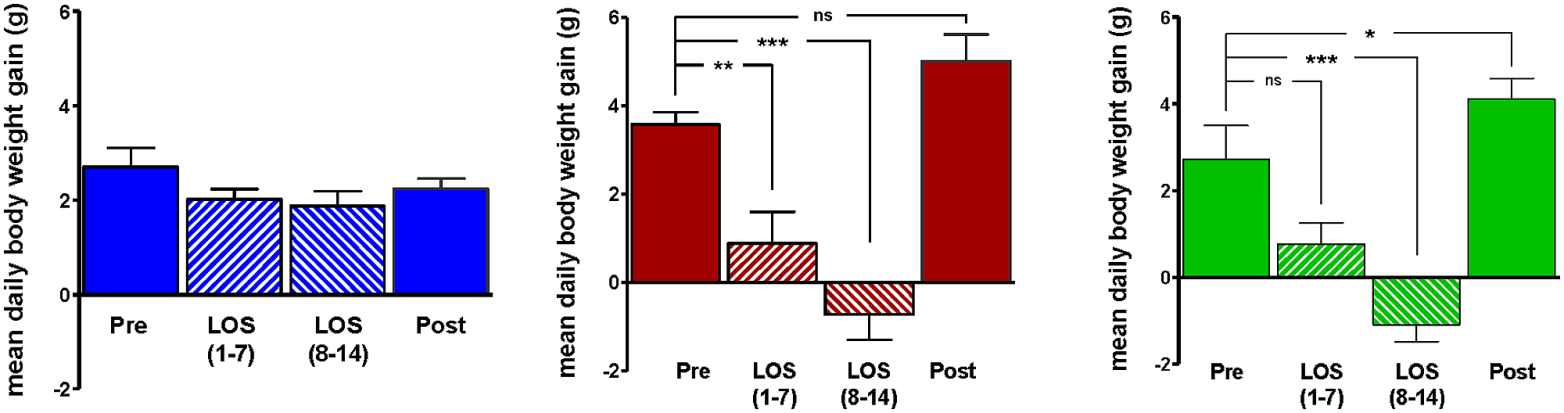
**Average daily weight gain in age-matched control rats fed standard rat chow (CHOW; left) or in DIO rats (middle) or DR rats (right), immediately prior to losartan treatment (Pre), days 1–7 (LOS 1–7) and days 8–14 (LOS 8–14) of losartan treatment and the final 7 days (days 18–24) following losartan treatment (Post).** Losartan attenuated body weight gain in DIO and DR animals as compared to control (*p < 0.05, **p < 0.01, ***p < 0.001; Tukey *post hoc* analysis).

**FIGURE 4:**
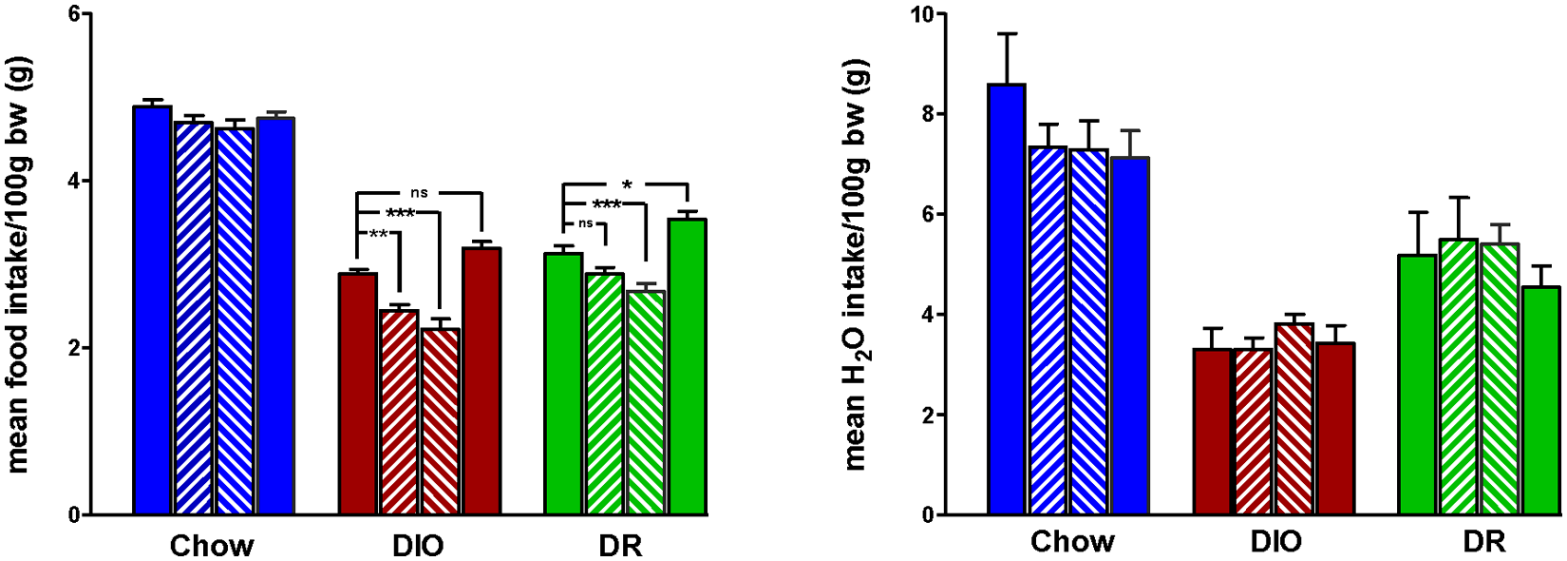
**Mean daily food (left panel) and water (right panel) intake for 7 days immediately prior to losartan treatment (Pre), days 1–7 (LOS 1–7) and days 8–14 (LOS 8–14) of losartan treatment and the final 7 days (days 18–24) following losartan treatment (Post).** Food intake was decreased in DIO and DR animals during losartan treatment compared to control values (*p < 0.05, **p < 0.01, ***p < 0.001; Tukey *post hoc* analysis) while losartan treatment had no effect on food intake in age-matched chow fed controlanimals compared to control food intake. Water intake was unaffected in all groups.

Body weight increased steadily in all three groups (DIO, DR, CHOW) during the 14 day control period (see **Figure 2**). However, during losartan administration rats consuming the HFD (DIO and DR) no longer demonstrated an increase in body weight while age-matched chow fed control animals continued to gain weight as they had previously to losartan administration (see **Figure 2**). As illustrated in **Figure 3**, mean daily body weight gain remained constant within all groups during the control period with age-matched chow fed control animals (CHOW) demonstrating a mean body weight gain of 2.8 ± 0.3 g/day (*n* = 8), DIO animals gaining 3.6 ± 0.3 g/day (*n* = 8) and DR animals gaining 2.1 ± 0.6 g/day (*n* = 8). Losartan administration abolished body weight gain in animals fed a HFD (DIO and DR, *p* < 0.001, repeated measures ANOVA) while chow fed animals continued to gain weight as they had prior to oral administration of losartan (*p* = 0.12, repeated measures ANOVA; see **Figures 2** and **3**). This decrease in daily body weight gain was accompanied by a decrease in food intake (see **Figure 4**) in the HFD fed animals (DIO and DR, *p* < 0.001, repeated measures ANOVA) while water consumption was not altered (*p* > 0.05, repeated measures ANOVA; see **Figure 4**). Following the removal of losartan both the DIO and DR animals again showed daily increases in body weight gain and food intake which were similar to control values (see **Figures 2–4**). Losartan administration was without effect on daily body weight gain, food intake or water consumption in the chow fed animals (see **Figures 2–4**).

## DISCUSSION

Here, we assessed the effect AT1R blockade with losartan on body weight and food intake in the rat DIO model of obesity. In line with previous findings (Machado et al., 2012; Sagae et al., 2013; Oliveira-Junior et al., 2014), we show that oral administration of losartan abolishes weight gain in DIO rats fed a HFD without influencing body weight in age-matched chow fed, animals. Our study, however, extends these findings to show that AT1R receptor blockade also prevents weight gain in animals fed a HFD but who were not obese (DR animals).

A role for AT1R in the development of DIO is supported by studies in AT1R knockout mice (Agtr1a^-/-^) that show an attenuation of diet induced body weight gain as compared to their wild type counterparts (Kouyama et al., 2005). Blockade of AT1R by a variety of pharmacological agents have also been shown to prevent HFD induced body weight gain (Sharieh Hosseini et al., 2014) or to impair body weight gain in DIO animals (Machado et al., 2012; Miesel et al., 2012; Sagae et al., 2013; Oliveira-Junior et al., 2014). An interesting finding of the present study is that AT1R blockade had a similar effect on weight gain in the DR animals but not in age-matched chow fed controls suggesting a role for the RAS on body weight gain only in animals receiving a HFD regardless of body weight and adiposity. This finding may in part be explained by the losartan-induced decrease in food intake, however, further studies to elucidate the mechanism(s) by which blockade of the RAS with losartan is able to prevent body weight gain in animals fed a HFD are warranted, though decreased leptin and increased adiponectin concentrations have already been proposed (Zorad et al., 2006; Weisinger et al., 2009b; Premaratna et al., 2012). In the present study, losartan administration did not change body weight gain in age-matched chow fed (CHOW) animals a finding that is at odds with previous work where administration of AT1R antagonists attenuated body weight gain in rats fed a normal diet (Zorad et al., 2006). These apparently contradictory findings may be due to the differences in duration of AT1R antagonist administration (14 days in the current study vs 18 week administration in the Zorad et al. (2006), study differences in strain of rat, or in the AT1R antagonist used. The fact that water consumption was not altered by losartan suggests that the losartan-induced drop in food intake observed in our study is not due to any secondary effect such as the satiety that results from increased water consumption.

The data we present here raises important questions that will require further studies to elucidate the mechanism(s) by which losartan exerts its protective effects on body weight gain in the response to a HFD. Future studies investigating alterations in energy expenditure as well as body composition may provide insights regarding specific mechanisms underlying losartan’s ability to inhibit weight gain. Analysis of circulating concentrations of the RAS and subsequent vascular and adipose reactivity to angiotensin II may also provide important information. Finally, measurement of circulating metabolic hormones (leptin, adiponectin,….) and hypothalamic neurotransmitters (αMSH, NPY, ….) prior to, during, and post losartan treatment may uncover mechanisms through which the AT1 receptor modulates these systems. Collectively, such future studies will likely elucidate the complexity through which the AT1 receptor activation participates in the pathophysiology of adiposity.

## CONCLUSION

Our data clearly demonstrates that oral losartan administration attenuates body weight gain in animals fed a HFD whether the animal is obese (DIO) or not (DR) while having no effect on body weight gain in age-matched chow fed animals. We hypothesize that angiotensin system blockade has a protective effect on body weight gain while on a HFD. Our data suggests that the value of losartan may extend beyond that of the treatment of hypertension and may be indicated as a suitable treatment for metabolic syndrome, the confluence of cardiovascular risk factors including hypertension, abdominal obesity, dislipidemia, and type 2 diabetes.

## Conflict of Interest Statement

The authors declare that the research was conducted in the absence of any commercial or financial relationships that could be construed as a potential conflict of interest.
